# Comparison of Visual Performance Between Two Diffractive Trifocal Intraocular Lenses

**DOI:** 10.3390/jcm14093128

**Published:** 2025-04-30

**Authors:** Gloria Segura-Duch, David Oliver-Gutierrez, Mar Arans, Susana Duch-Tuesta, Carlos Carpena-Torres, Gonzalo Carracedo, David Andreu-Andreu

**Affiliations:** 1 Verte-ICO Oftalmología, 08006 Barcelona, Spain; glo.segura.duch@gmail.com (G.S.-D.); davidoliguti@gmail.com (D.O.-G.); marans@verte.es (M.A.); dandreu@verte.es (D.A.-A.); 2 Ocupharm Research Group, Department of Optometry and Vision, Complutense University of Madrid, 28037 Madrid, Spain; ccarpena@ucm.es (C.C.-T.); jgcarrac@ucm.es (G.C.)

**Keywords:** diffraction, multifocality, defocus curve, visual acuity, trifocal intraocular lenses

## Abstract

**Background/Objectives**: This study aimed to compare the visual outcomes of two diffractive trifocal intraocular lenses (IOLs): the Bi-Flex Liberty 677MY (Medicontur; Zsámbék, Hungary) and the FineVision POD F (BVI Medical; Waltham, MA, USA). **Methods**: A prospective study with a 3-month follow-up was conducted. A total of 62 patients were divided into two groups according to the type of lens implanted: 31 patients with the Liberty lens (61.1 ± 6.4 years) and 31 patients with the Finevision lens (61.9 ± 6.8 years). Three measurement sessions were conducted (baseline, 1 month, and 3 months). These sessions included measurements of the subjective refraction, visual acuity, and defocus curve. Both eyes of each patient were operated on and included in the statistical analysis. **Results**: Three months after surgery, monocular visual acuity with the Liberty lens was statistically greater than with the Finevision lens at defocus values of −2.00 D (50 cm) and −2.50 D (40 cm) (*p* < 0.01). In this regard, the near visual acuity results (40–50 cm) with the Liberty lens showed greater variability compared to those of the Finevision lens. Binocularly, however, the Finevision lens demonstrated a statistically significant improvement in visual acuity than the Liberty lens at a defocus of −1.50 D (67 cm) (*p* = 0.01). Both IOLs provided visual acuities better than 0.20 logMAR for a defocus range from distance (0.00 D) to near (−3.50 D). **Conclusions**: Future studies are needed to investigate which patient ocular parameters could predict improved near vision with the Liberty lens or intermediate vision with the Finevision lens.

## 1. Introduction

Advancements in intraocular lens (IOL) implantation for cataract surgery over the last decade have enabled a growing number of patients to achieve independence from spectacles, thereby improving their quality of life [1]. Monofocal IOLs, the most commonly implanted lenses worldwide, correct refractive error exclusively for distance vision [2,3]. Consequently, patients with monofocal IOLs require spectacles for intermediate and near daily activities. To eliminate postoperative dependence on spectacles, the latest generation of multifocal IOLs is designed to create multiple focal points on the retina, ensuring clear and stable vision at all distances [4,5].

Despite nearly 100 multifocal IOLs with different materials and optical designs being available, it is estimated that they are implanted in only 10–15% of cataract surgeries [6]. In recent years, surgeons increasingly favored extended depth-of-focus (EDOF) and trifocal designs among the many available options [7,8]. In the case of trifocal IOLs, the focus of the current study, multifocality, is achieved by converging light into three primary focal points: distance, intermediate, and near. This trifocality not only ensures high visual quality at these three distances but also provides clear vision across the entire range of vision [9,10,11,12].

Within trifocal designs, there is a wide variety of IOLs available on the market [8]. One such lens is the Bi-Flex Liberty 677MY (Medicontur; Zsámbék, Hungary), which presents a diffractive design in the central 3 mm and an aspheric trifocal design in the periphery of its 6-mm optical zone. In a case series study performed by García-Bella et al. [13], this lens demonstrated adequate visual function at all distances. However, its clinical validation has not been carried out in comparison with other available multifocal IOLs.

For this reason, the purpose of this study was to compare the visual outcomes of the Liberty lens with those of the FineVision POD F (BVI Medical; Waltham, MA, USA). The Finevision lens, like the Liberty lens, features an aspheric trifocal design with a 6-mm optical zone, within which there is a central diffractive pattern whose diameter is not specified by the manufacturer. The Finevision lens was chosen as the control because, as the first diffractive trifocal IOL on the market, it remains one of the most widely implanted lenses, with its visual efficacy extensively reported in the scientific literature [14,15,16].

## 2. Materials and Methods

### 2.1. Study Design and Patients

A prospective study with a 3-month follow-up was conducted. A total of 62 patients were included in the study and divided into two groups according to the type of lens implanted: 31 patients with the Liberty lens and 31 patients with the Finevision lens. The first 31 patients were consecutively implanted with the Finevision lens, while the last 31 were implanted with the Liberty lens. Three measurement sessions were conducted: preoperative (baseline) and at 1- and 3-months post-surgery, within a visit margin of ±1 week. These sessions included measurements of subjective refraction, visual acuity, and defocus curve. Both eyes of each patient were operated on and included in the statistical analysis.

The inclusion criteria were presbyopes aged between 50 and 80 years, presence of any degree of cataracts or a clear crystalline lens, availability to attend all postoperative follow-up visits, absence of ocular pathology contraindicating trifocal lens implantation, and a personal desire to achieve trifocality following crystalline lens surgery. The exclusion criteria were unstable general health (e.g., cerebrovascular or neurodegenerative diseases), ocular pathology contraindicating trifocal lens implantation, prior refractive surgery or lens implantation, preoperative refractive astigmatism higher than 1.25 D, and intraoperative complications such as significant vitreous loss, pupillary trauma, zonular damage, and capsular rupture or injury.

The principles of Good Clinical Practice guidelines, current Spanish and European legislation, and the Declaration of Helsinki were followed. All procedures were performed at the Innova Ocular ICO ophthalmology clinic (Barcelona, Spain). The study protocol was approved in advance by the Clinical Research Ethics Committee of the *Hospital Clínic de Barcelona* (code HCB/2023/0469; approval date: 19 January 2024). All participants voluntarily enrolled in the study after signing the study’s informed consent form, and they were free to leave the study at any time, with their postoperative revisions not being affected.

### 2.2. Surgical Procedure and Intraocular Lenses

All cataract surgeries were performed by two experienced ophthalmologists, following the same protocol for all patients. Topical anesthesia was used in all cases. The phacoemulsification technique was carried out using a standard procedure, involving a 2.2 mm corneal incision for aspiration and irrigation of the crystalline lens cortex. Subsequently, the intraocular lens was implanted through a hydraulic injector, specific to each lens type, following the manufacturer’s instructions.

Depending on the assigned group, patients were implanted with the Liberty lens or the Finevision lens, whose technical characteristics are detailed in Table 1. The lens power was calculated based on subjective refraction, optical biometry data, and the Olsen formula, targeting a postsurgical spherical refraction of −0.25 D.

### 2.3. Preoperative and Postoperative Measurements

High-contrast visual acuity (100%) was measured under photopic conditions using the Early Treatment Diabetic Retinopathy Study (ETDRS) test [17], incorporated into the Optonet digital display (Optonet Ltd.; Warrington, UK), for both distance vision (4 m) and near vision (40 cm). Distance and near visual acuities were measured monocularly, both uncorrected and corrected.

The monocular and binocular defocus curves were measured using the Multifocal Lens Analyzer app (Qvision; Almeria, Spain) for the iPad tablet (Apple Inc.; Cupertino, CA, USA). The procedure involved measuring high-contrast visual acuity using the ETDRS test for defocus values ranging from +1.00 D to −4.00 D, in 0.50 D steps. This allows the assessment of the patient’s visual acuity at different distances [18]. The defocus curves were measured without compensating for the residual refractive error of the patients.

### 2.4. Statistical Analysis

Statistical analysis was performed using SPSS Statistics 23 software (IBM, Chicago, IL, USA). The normality of the distributions was assessed with the Shapiro–Wilk test. Statistical comparisons of the different variables were performed between the two implanted lenses for each follow-up visit (baseline, 1 month, and 3 months). For qualitative variables, the Chi-squared test was used. For quantitative variables, the Mann–Whitney U test was applied in cases of non-normal distribution, and the Student’s *t*-test for independent samples was used in cases of normal distribution.

A significance level of 95% (*p* < 0.05) was established for all statistical tests. The analyzed variables were gender, age, sphere, cylinder, and visual acuities (uncorrected distance, corrected distance, uncorrected near, corrected near, and defocus curves). The total number of patients assigned to each group was determined based on previous scientific literature studies analyzing the visual outcomes of the trifocal IOLs used in the current study [13,14,15,16].

## 3. Results

The demographic and refractive characteristics of the patients assigned to each study group are detailed in Table 2. No statistically significant differences were found between the patients implanted with Liberty and Finevision lenses in terms of age, gender, or preoperative refractive parameters (sphere and cylinder) (*p* ≥ 0.05).

**Figure 1:**
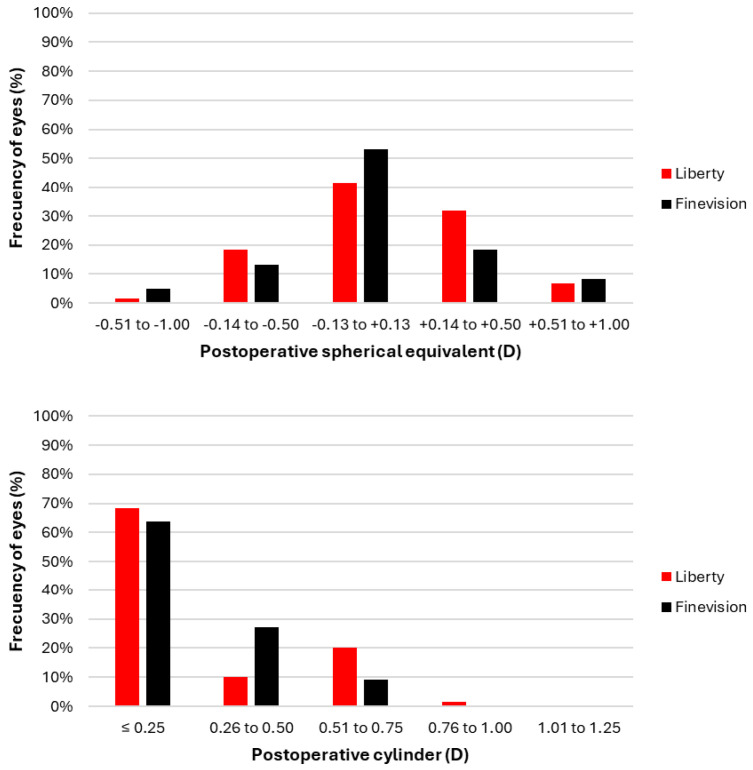
Refractive outcomes for both intraocular lenses 3 months post-surgery. The y-axes represent, in terms of frequency, the number of eyes that achieved postoperative values of spherical equivalent (top) or cylinder (bottom) within the ranges indicated on the x-axes.

Figure 1 shows the refractive outcomes in terms of spherical equivalent and cylinder for both lenses, 3 months after surgery. A total of 91.7% of patients implanted with the Liberty lens and 85.0% of those with the Finevision lens achieved a spherical equivalent within ±0.50 D. Regarding cylinder values, 78.3% of patients had values ≤ 0.50 D with the Liberty lens, compared to 90.9% with the Finevision lens. The mean residual sphere was +0.09 ± 0.32 D for the Liberty lens and +0.08 ± 0.44 D for the Finevision lens, with no significant differences between the two lenses (*p* = 0.953). Similarly, the mean residual cylinder was −0.32 ± 0.28 D for the Liberty lens and −0.27 ± 0.29 D for the Finevision lens, also showing no significant differences between them (*p* = 0.342).

Regarding monocular visual acuity outcomes (Table 3), a statistically significant improvement of 0.02 ± 0.05 logMAR was observed in the uncorrected near visual acuity of patients implanted with the Finevision lens compared to the Liberty lens 1 month after surgery (*p* < 0.01). However, these differences disappeared 3 months post-surgery (*p* = 0.21). The remaining visual acuity measurements (uncorrected distance, corrected distance, and corrected near) showed no significant differences between the lenses (*p* ≥ 0.05).

**Figure 2:**
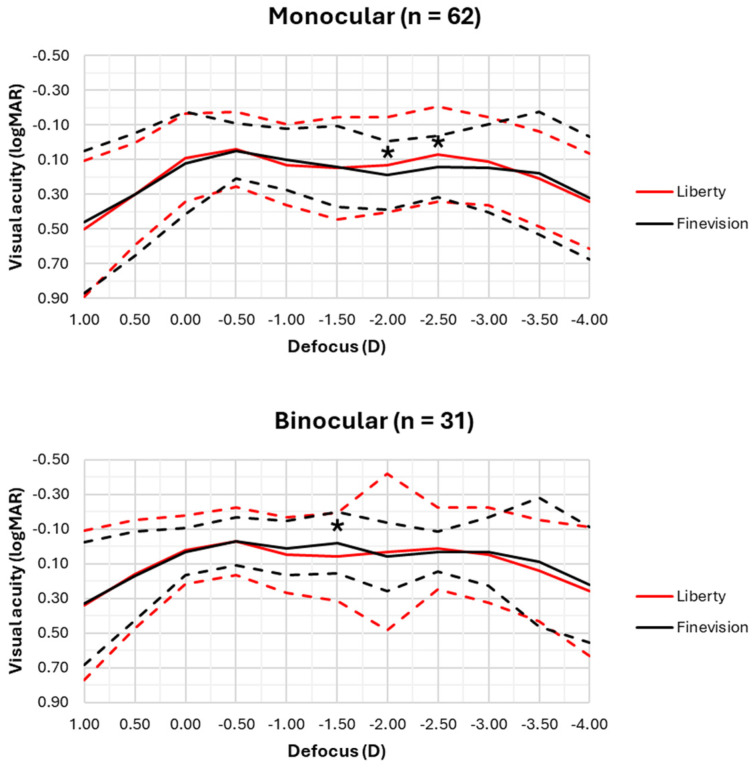
Monocular and binocular defocus curves for both intraocular lenses, 3 months post-surgery. The dashed lines represent the 95% confidence intervals (CI) of visual acuity at each defocus level. The 95% CI is mathematically defined as 2.77 × the standard deviation and represents the range of visual acuity that can be expected for 95% of the patients implanted with each of the lenses. * *p* < 0.05, Mann–Whitney U test.

Finally, Figure 2 shows the monocular and binocular defocus curves measured 3 months after surgery with each of the implanted lenses. Monocularly, visual acuity with the Liberty lens was significantly better than with the Finevision lens at defocus values of −2.00 D (50 cm) and −2.50 D (40 cm) (*p* < 0.01). Binocularly, however, the Finevision lens demonstrated a more statistically significant improvement in visual acuity than the Liberty lens at a defocus of −1.50 D (67 cm) (*p* = 0.01). It should be noted that the maximum monocular and binocular visual acuity for both lenses was achieved at a defocus of −0.50 D (2 m).

## 4. Discussion

This study evaluated, for the first time, the visual efficacy of the Liberty lens implantation compared to another diffractive trifocal IOL, the Finevision lens. Both IOLs demonstrated adequate visual acuity at all distances, with some differences in the intermediate-to-near vision range, which will be discussed in this section.

It should be noted that no significant differences were observed between the two lenses in distance and near visual acuity measured by the ETDRS test on the digital display (see Table 3). However, differences in intermediate and near vision were found in the defocus curves (see Figure 2). This discrepancy could be associated with the fact that defocus curves measured using the app are semi-automated, which would reduce inter-examiner bias introduced when optotype readings are guided by examiners [19]. This highlights the importance of measuring visual acuity after surgery using a single examiner or automated devices in this type of clinical study.

Regarding monocular defocus curves, the implantation of the Liberty lens demonstrated near visual acuity (at 40–50 cm) up to three letters (0.06 logMAR) better than the Finevision lens. However, these differences were not found under binocularity. The fact that the Liberty lens exhibited better near visual acuity under monocular conditions but not under binocular conditions may be attributed to the high variability observed in these measurements. This suggests that while some eyes implanted with the Liberty lens may achieve better near visual acuity, poorer acuity in the contralateral eye could prevent this improvement from being perceived under binocular conditions.

On the other hand, under binocularity, patients implanted with the Finevision lens showed intermediate visual acuity (at 67 cm) that was four letters (0.08 logMAR) superior to those implanted with the Liberty lens. This increased intermediate visual acuity could be associated with binocular summation [20], given that the variability in visual acuity was lower with this lens in both monocular and binocular conditions.

Although the results under monocular and binocular conditions suggest that the Liberty lens may provide better near visual acuity and the Finevision lens better intermediate visual acuity, these differences may not be considered clinically relevant, as the mean differences in visual acuities between both lenses were below 0.10 logMAR. In this sense, it remains unclear with both lenses as to which ocular parameters of the patients (pupil size, preoperative aberrometry, kappa angle, etc.) could predict better postoperative visual quality at near or intermediate distances and whether having these parameters truly represents a clinically relevant improvement for the patients.

According to the new functional classification of IOLs proposed by Fernandez et al. [21], which is based on the profile of monocular defocus curves, the Liberty lens would be classified as a *full range of field (FULL-RoF) smooth*, while the Finevision lens would be categorized as *FULL-RoF steep*. The fact that both lenses are classified as *FULL-RoF* indicates that they provide a defocus range from distance (0.00 D) to near (−3.50 D) with visual acuity better than 0.20 logMAR. However, the classification of the Liberty lens as *smooth* means it has a variation in visual acuity within this defocus range between 0.05 and 0.13 logMAR, whereas the variation with the Finevision lens exceeds 0.13 logMAR (*steep*). The defocus curve profiles align with those previously published in other studies for both the Liberty lens [13] and the Finevision lens [14,15,16], which are typical of trifocal IOLs with higher addition [21].

This study presented some limitations that should be highlighted. Firstly, aside from visual acuity, other parameters of visual function (e.g., contrast sensitivity, halo size, or associated symptoms) were not evaluated, which could provide further insights into the visual performance of the implanted IOLs. Secondly, the lack of a quality-of-life questionnaire represents another limitation, as it could have provided valuable information about the subjective impact of the lenses on patients’ daily lives, beyond visual parameters alone. Thirdly, it was not possible to analyze other predictive factors of visual quality that could help identify, in general terms, which type of patient might benefit more from better near vision with the Liberty lens or better intermediate vision with the Finevision lens. Moreover, it is important to note that the patients, the ophthalmology center, and the investigators in this study had their own socioeconomic, clinical, and professional particularities, which is why the results should be extrapolated to a general population with caution.

## 5. Conclusions

Both diffractive trifocal IOLs demonstrated adequate visual acuity (better than 0.20 logMAR) at all distances. However, the Liberty lens showed a statistically significant improvement in near vision compared to the Finevision lens, although this was accompanied by greater variability, making it difficult to generalize these results to all future implanted patients. As an example of this variability, the Finevision lens was observed to achieve better intermediate visual acuity under binocular conditions. These findings should be interpreted with caution, considering that although there were statistically significant differences in visual acuity between both lenses, their clinical relevance may be limited, as the mean differences were below 0.10 logMAR. Therefore, future studies are needed to investigate which patient ocular parameters could predict improved near vision with the Liberty lens or intermediate vision with the Finevision lens.

## Figures and Tables

**Table 1:** Technical characteristics of the intraocular lenses implanted in the study.

Parameter	Liberty	Finevision
Technical name	Bi-Flex Liberty 677MY	FineVision POD F
Manufacturer	Medicontur (Zsámbék, Hungary)	BVI Medical (Waltham, MA, USA)
Material	25% Hydrophilic	26% Hydrophilic
Overall diameter (mm)	13.00	11.40
Optic diameter (mm)	6.00	6.00
Diffractive diameter (mm)	3.00	Unspecified
Optic design	Biconvex aspheric trifocal	Biconvex aspheric trifocal
Haptic design	Posterior vaulting fenestrated C-loop (0° angulation)	4-closed C-loops and posterior angulated haptic
Refractive index	1.46	1.46
Abbe number	58	58
A constant (SRK/T)	118.9	118.8
Addition (IOL plane)	+1.75 D, +3.50 D	+1.75 D, +3.50 D
Filtration	UV, blue light	UV, blue light

Information provided by the manufacturers.

**Table 2:** Demographic and preoperative refractive characteristics of the patients included in the study.

Variable	Liberty	Finevision	*p*-Value
Participants (n)	31	31	-
Eyes analyzed (n)	62	62	-
Gender (male, %)	29.0%	32.3%	0.68
Age (years)	61.1 ± 6.4	61.9 ± 6.8	0.47
Sphere (D)	0.65 ± 1.93	0.81 ± 2.13	0.41
Cylinder (D)	−0.81 ± 1.01	−0.60 ± 0.59	0.66

The statistical comparison was performed between both lenses.

**Table 3:** Monocular visual acuity outcomes for both intraocular lenses at baseline, 1 month, and 3 months post-surgery.

Variable	Lens	Baseline	1 Month	3 Months
UDVA (logMAR)	Liberty	0.56 ± 0.06	0.08 ± 0.07	0.09 ± 0.08
	Finevision	0.53 ± 0.09	0.08 ± 0.09	0.10 ± 0.10
	*p*-Value	0.60	0.49	0.98
CDVA (logMAR)	Liberty	0.05 ± 0.08	0.01 ± 0.03	0.02 ± 0.04
	Finevision	0.07 ± 0.11	0.03 ± 0.07	0.02 ± 0.06
	*p*-Value	0.22	0.43	0.94
UNVA (logMAR)	Liberty	-	0.03 ± 0.05	0.01 ± 0.02
	Finevision	-	0.01 ± 0.05	0.02 ± 0.06
	*p*-Value	-	< 0.01 *	0.21
CNVA (logMAR)	Liberty	-	0.00 ± 0.02	0.00 ± 0.02
	Finevision	-	0.01 ± 0.05	0.02 ± 0.06
	*p*-Value	-	0.73	0.08

UDVA: Uncorrected distance visual acuity; CDVA: Corrected distance visual acuity; UNVA: Uncorrected near visual acuity; CNVA: Corrected near visual acuity. The statistical comparison was performed between both lenses. * *p* < 0.05, Mann–Whitney U test.

## Data Availability

The raw data supporting the conclusions of this article will be made available by the authors on reasonable request.
